# High-Flow vs. Low-Flow Nasal Cannula in Reducing Hypoxemic Events During Bronchoscopic Procedures: A Systematic Review and Meta-Analysis

**DOI:** 10.3389/fmed.2022.815799

**Published:** 2022-02-24

**Authors:** Fotios Sampsonas, Vasileios Karamouzos, Theodoros Karampitsakos, Ourania Papaioannou, Matthaios Katsaras, Maria Lagadinou, Eirini Zarkadi, Elli Malakounidou, Dimitrios Velissaris, Grigorios Stratakos, Argyrios Tzouvelekis

**Affiliations:** ^1^Respiratory Medicine Department, University Hospital of Patras, Patras, Greece; ^2^Intensive Care Unit, University Hospital of Patras, Patras, Greece; ^3^Internal Medicine Department, University Hospital of Patras, Patras, Greece; ^4^Interventional Pulmonology Unit, First Respiratory Medicine Department of the National Kapodistrian University of Athens, Athens, Greece

**Keywords:** high-flow nasal cannula, low-flow nasal cannula, EBUS TBNA, hypoxemia, meta-analysis, bronchoscopy

## Abstract

**Introduction:**

High-flow nasal cannula (HFNC) oxygenation method has been proven to be successful in oxygenation of patients with respiratory failure and has exhibited clinical superiority compared to low-flow nasal cannula (LFNC).

**Methods:**

We performed a systematic review and meta-analysis to evaluate the potential favorable impact of HFNC oxygenation during bronchoscopy and related procedures like endobronchial ultrasound-transbronchial needle aspiration. Only randomized control trials (RCTs) were included in the meta-analysis.

**Results:**

Six randomized control trials with 1,170 patients were included in this meta-analysis. Patients who underwent bronchoscopy with the use of high-flow nasal cannula experienced less hypoxemic events/desaturations, less procedural interruptions and pneumothoraxes compared to patients under low-flow nasal cannula treatment. This beneficial effect of HFNC in hypoxemic events was persistent 10 min after the end of procedure.

**Conclusion:**

The high-flow nasal cannula (HFNC) oxygenation method could reduce hypoxemic events and related peri- and post-bronchoscopic complications.

## Introduction

Oxygen supplementation therapy through high-flow nasal cannula (HFNC) has evolved over the last decade to an integral component of the management of patients with respiratory failure in multiple clinical scenarios (1). It is a noninvasive mechanical respiratory support modality developed to deliver a humidified and heated (37°C) mixture of oxygen and air. Flow rates can vary from 10 to 80 L/min, where a continuous positive airway pressure (CPAP) effect can also develop (1, 2) by overcoming patient peak inspiratory flow rates, decreasing and washing out the anatomical dead space and, therefore, preventing alveolar collapse and decrease of dynamic compliance (3). Specially developed nasal prongs are used to deliver the oxygen flow, with inspired oxygen fraction (FiO_2_) ranging from 0.21 to 1 (1–4). HFNC can also successfully compensate increased inspiratory flow rates developed by a patient, providing a predictable and accurate FiO2 that can reach 1 (5). At the same time, it improves sputum expectoration by maintaining mucociliary clearance even at high flow rates due to humidification and heating of breathing-inspired air mixture (5) and finally reduces the work of breathing and preserves upper airway patency (4, 6).

Fiber optic bronchoscopy (FOB) is a diagnostic and interventional tool widely used in patients with a variety of respiratory diseases (7). Endobronchial ultrasound-guided transbronchial needle aspiration (EBUS-TBNA) has been also established as the core component for lung cancer diagnostic approach, by aspirating mediastinal lymph nodes and related pathologies (8). Periprocedural hypoxemia and respiratory failure development do not represent a rare complication of bronchoscopy, especially in sedated elderly patients with underlying comorbidities (9). Oxygen supplementation using a low-flow nasal cannula (LFNC) is advisable to be employed during the procedure in order to compensate for the development of hypoxemia and respiratory failure (7). Hypoxemia during bronchoscopy is associated with periprocedural complications like arrythmias and, thus, should be generally avoided and, if encountered, must be reverted (7).

In view of these advantages of HFNC over LFNC, and having in mind the increased need for accurate, safe, and prompt diagnosis of a plethora of lung diseases with the use of bronchoscopy/EBUS in patients with multiple comorbidities, we performed a systematic review and meta-analysis to address the possible favorable impact of HFNC administration in the development of respiratory failure during these bronchoscopic procedures.

## Methods

### Study Criteria

Only randomized controlled trials (RCTs) were included in our study. Our study population comprised of hospitalized patients and outpatients who underwent diagnostic bronchoscopic procedures (FOB and/or EBUS-TBNA). Intensive care and pediatric patients were excluded. All studies comparing HFNC with LFNC during bronchoscopic procedures were included. Studies comparing different types of high-flow devices and/or different interfaces were excluded.

### Search Strategy and Article Selection

**Figure 1:**
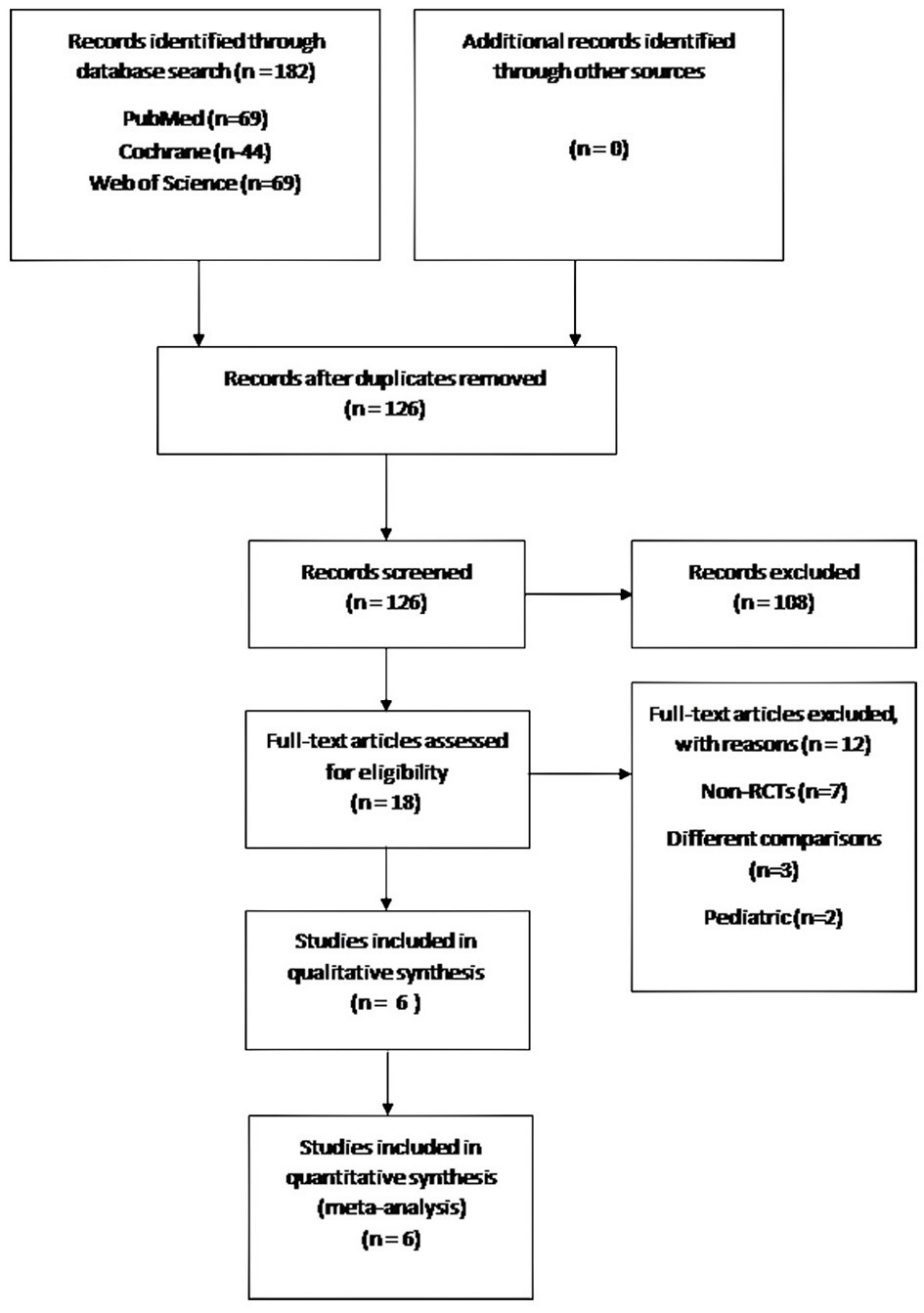
Study flow chart.

The Preferred Reporting Items for Systematic Reviews and Meta-Analysis (PRISMA) statement (10) was used for this study. Two researchers (FS and VK) performed an independent thorough search of the PubMed/Medline, EMBASE, Web of Science, and Cochrane databases from inception to December 2021 using the keywords “high flow oxygen,” “high flow nasal oxygen,” “HFNC,” “bronchoscopy,” and “Endobronchial Ultrasound/EBUS.” No publication date restrictions were applied, and only articles in English were included. After removing all duplicates, two researchers (FS and VK) independently reviewed all abstracts, and all eligible articles were retrieved for further assessment. Disagreements were resolved by consensus with a senior author (AT). The process of article selection is presented in the PRISMA flow chart (Figure 1).

### Data Extraction

Data extraction was performed independently by two authors (FS and VK) while two other authors (TK and AT) examined the two data sets for discrepancies. The extracted data included first author name, year of publication, country, chronicity, study design, procedure type, inclusion/exclusion criteria, sample size, mean age, gender, exposure/control group, and outcome data. In the study of Douglas et al. (11), the authors decided to perform a per protocol analysis after reporting results of an intention-to-treat analysis. We included in our study the more conservative intention-to-treat analysis results.

### Outcomes

Our primary outcome was desaturation events, defined as an oxygen saturation (SpO_2_) drop below 90% at least once during the bronchoscopic procedure. Secondary outcomes were the lowest value of SpO_2_ during the procedure, hypoxemia duration, procedure duration, partial pressure of carbon dioxide (PCO_2_) at the end of the procedure, respiratory rate (RR), heart rate (HR), mean arterial pressure (MAP) and SpO_2_ 10 min after the end of the procedure. Furthermore, incidence of pneumothorax and procedural interruption were also examined.

### Quality Assessment

Quality assessment was also carried out independently by two authors (FS and VK) using the Risk of Bias tool (RoB 2) for RCTs (12). The authors classified the included studies as low, moderate, or high/serious risk. Risk of bias visualization was performed in R using the Risk-of-bias VISualization (robvis) package (13).

### Statistical Analysis

A pairwise meta-analysis was performed in R version 3.6.1 using the packages “meta” and “metafor.” A random effects model was used for all outcomes. Risk ratio and mean difference were selected as the measure of effect for binary and continuous outcomes, respectively. *Q* and *I*^2^ statistics were used to explore and measure heterogeneity, respectively. *I*^2^ = 0–40% suggests not important heterogeneity, *I*^2^ = 30–60% suggests moderate heterogeneity, *I*^2^=50–90% suggests substantial heterogeneity, and *I*^2^ = 75–100% suggests considerable heterogeneity. In studies with data not normally distributed, the mean and standard deviation were estimated using sample size, median, and interquartile range (IQR) provided that skewness was acceptable (14, 15). Publication bias detection using a funnel plot was not carried out, since in our analysis we included < 10 studies, and the test is not powered for such a small sample size.

## Results

### Study Characteristics

Six studies were included in the qualitative assessment with a total of 1,170 patients (11, 16–20). All patients were outpatients and/or hospitalized patients but not intubated or patients treated in the intensive care unit. In three studies, patients underwent fiberoptic bronchoscopy (FOB) ± bronchoalveolar lavage (BAL) or biopsy; and in three studies, EBUS-TBNA was performed. In four studies, intravenous sedation was administered and in two only topical sedation. All six studies were included in the quantitative analysis (11, 16–20). Study characteristics are summarized in Table 1.

**Table 1:** Characteristics of analyzed studies.

	**First author**	**Type of study**	**Country/Chronicity**	**Procedure/Access route**	**Inclusion criteria**	**Exclusion criteria**	**Sample size (C/I)**	**Age (C/I) / F. gender (C/I)**	**Outcomes**	**Sedation**	**Control/Intervention group**	**Main results**
1	Ben-Menachem et al. (17)	RCT	Australia	FOB with transbronchial lung biopsy	Adult, Transplant recipients	<18 years, non-lung transplant patients, cardiovascular or respiratory failure, reduced level of consciousness, pregnancy, significant aspiration risk, need for a laryngeal mask airway or endotracheal intubation, unable to have sedation with propofol or unsuitable for HFNC such as recent nasal surgery or a base of skull defect or fracture	76 patients	55.8/54.9	Procedure duration, Dose of Sedative agents, Bispectral Index, Satisfaction score, Duration of desaturation, Pneumothorax	Preprocedure: Nebulized 2% lidocaine and midazolam 1 to 3mg i.v. Procedure: propofol and alfentanil	C: 4-10L LFNC	The proportion of patients with desaturation, SpO2 < 90%, was lower in the HFNO group; 16.2% vs. 69.2% in the LFNO group (P < 0.001), The Duration of desaturation was Higher in LFNC, Anesthetist was more satisfied with HFNC.
			May 2018 to May 2019	Oral			C:39	C: 10			I: 30-50L HFNC	
							I:37	I: 15				
2	Douglas et al. (11)	RCT	Australia	EBUS	Adult, able to give informed consent, sedation planned, and English speaking	<18 years, unable to consent, trachea intubated or requiring intubation for procedure, pregnant, active nasal bleeding, or base of skull fracture	60 patients	63.4/62.8	Proportion of patients experiencing desaturation below 90%, oxygen saturation, duration of hypoxia, end-procedure end-tidal CO2, satisfaction score, number of interruptions, arrhythmia, myocardial ischaemia and cardiac arrest	Preprocedural: topical lidocaine 2% Procedural: midazolam, opioids and/or propofol	C: 10-15L LFNC	Spo2 < 90% in Intension to treat analysis revealed no difference (4/30 vs. 10/30). In the Per protocol analysis significant difference 4/31 vs. 10/29, The SpO_2_ following pre-oxygenation was significantly higher in the HFNO The median lowest SpO_2_ observed during the procedure in the HFNO group was significantly higher
			14 February 2017–23 May 2017	No information			C:30	C: 11			I: 30-70L FiO_2_: 100% HFNC	
							I:30	I: 11				
3	Irfan et al. (19)	RCT	UK	EBUS-TBNA	Adult, saturation ≥90% on air, Being able to breathe spontaneously throughout the procedure	Cardiorespiratory failure, recent myocardial infarction within 6 wk, long-term oxygen therapy, tracheostomy, noninvasive ventilation, nasal or nasopharyngeal disease, inability to give informed consent, dementia, hepatic or end-stage renal disease, pregnancy	40 Patients	64.5/61.9	Primary end-point: drop in the oxygen saturations from the start of the procedure Secondary end-points: changes in venous blood CO2 lowest oxygen saturation, Changes in end tidal CO2, requirement for intubation, overall experience on a visual analog scale (VAS)	Preprocedural: local anesthesia Procedural: midazolam and alfentanil	C: LFNC	Primary outcome: Oxygen desaturation was statistically significant with a difference of 7.7 percentage points Secondary outcome: The lowest oxygen saturation was also statistically significant with a difference of −9.2.
			No Information	No information			C:2	No information			I: HFNC	
							I: 20					
4	Longhini et al. (16)	RCT	Italy	FOB with BAL	Adult (aged ≥18 years), outpatients	life-threatening arrhythmia, recent myocardial infarction, oxygen therapy or home mechanical ventilation, pulmonary emphysema, history of spontaneous pneumothorax, recent thoracic surgery, presence of skin lesions on the chest, tracheostomy, chronic elevation and/or paralysis of a hemidiaphragm, inability to express an informed consent, consent withdrawal, presence of morbid obesity, inclusion in other research protocols.	36 patients	No information	PaO2 at the end of FOB with BAL, the lowest peripheral saturation of oxygen (SpO2) and the number of oxygen desaturations, the changes of end-expiratory lung impedance (EELI) and tidal impedance assessed by electrical impedance tomography (EIT), the effects on diaphragm function assessed by ultrasound	Preprocedural: topical lidocaine 2% Procedural: topical lidocaine 2%	C: LFNC	10 (56%) patients had one or more episodes of desaturation in the LFNC group, while only 2 patients (11%) in the HFNC group
			September 2019 to February 2020	Oral			C:18	C: 6			I: HFNC 60L starting at 0.21 FiO2	
							I:18	I: 3				
5	Ucar et al. (18)	RCT	Turkey	EBUS-TBNA	Adult patients	Body mass index (BMI) higher than 30, tracheostomy, nasal or nasopharyngeal disease, difficulty in communicating, pregnancy	170 patients	57.8/57.5	Desaturation from baseline, Heart Rate. Blood Pressure immediate and 10' after procedure, patient self-reported comfort	Preprocedural: topical lidocaine 2% Procedural: midazolam	C: LFNC of similar FiO2	5 (6%) one or more episodes of desaturation in HFNC, 26 (31%) in ST group
			2018–2019	Oral			C: 85	C:55			I: HFNC FiO2 40%, 35lt	
							I: 85	I: 56				
6	Wang et al. (20)	RCT	China	FOB + BA	Adult and indication for diagnostic bronchoscopy	SpO2 < 90% on room air, platelet count < 60 × 10^9^/L, and nasopharyngeal obstruction or blockage	788 patients	59/58	The primary endpoint was the proportion of patients with a single moment of SpO2 < 90%. The secondary endpoint was the duration of Bronchoscopy. Other endpoints were durationof SpO2 < 90% and the proportion of patients with procedural discontinuation	Preprocedural: topical lidocaine 2% Procedural: topical lidocaine 2%	C: LFNC 6L	The proportion of patients with a single moment of SpO2 < 90% during bronchoscopy in the HFNC group was significantly lower than that in the LFNC. The lowest SpO2 during bronchoscopy and 5 min after bronchoscopy in the HFNC group was significantly higher than that in the LFNC group.
			November 2015 to October 2019	Nasal			C: 396	C: 174			I: HFNC 50L	
							I: 392	I: 188				

*RCT, randomized control trial, FOB, fiberoptic bronchoscopy, HFNC, High Flow Nasal Canula, LFNC, Low flow nasal canula, EBUS, Endobronchial Ultrasound, FiO_2_, fraction of inspired oxygen, SpO_2_, peripheral oxygen saturation*.

### Quality Assessment

**Figure 2:**
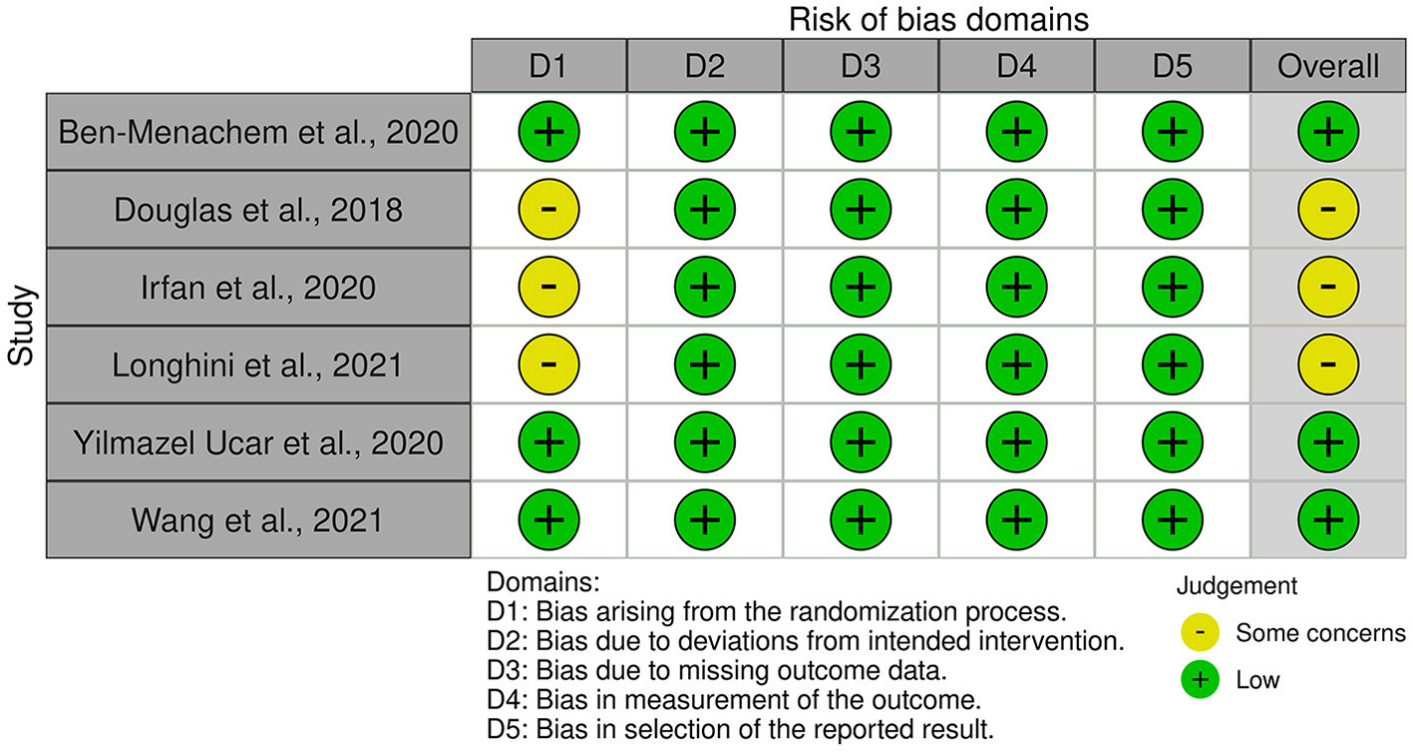
Risk of bias for randomized control trials.

**Figure 3:**
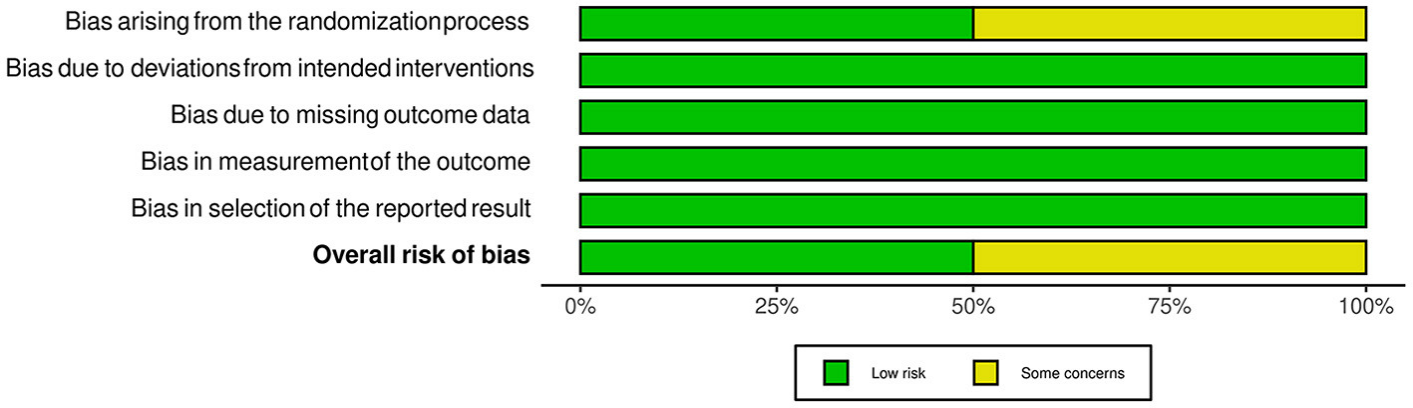
Overall risk of bias for randomized control trials.

For RCTs, we used the ROB 2 tool to evaluate the risk of bias in the included studies. Three studies were judged as studies with some concerns in the first domain. Two of them exhibited imbalances in baseline characteristics (11, 19), and in the third trial, age was not reported (16). The results are summarized in Figures 2, 3.

### Incidence of Desaturation During Bronchoscopy

**Figure 4:**
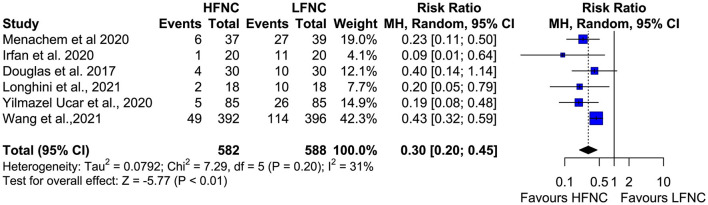
Meta-analysis of desaturation events (SpO2 < 90%) in patients receiving HFNC compared to LFNC in patients undergoing bronchoscopic procedures.

All six studies measured the incidence of desaturation (SpO_2_ < 90% at least once during the procedure) during bronchoscopy (11, 16–20). The incidence of desaturation was statistically lower in patients who received support with HFNC during bronchoscopic procedures (RR = 0.3; 95% CI = 0.2,0.45; *p* < 0.01; *I*^2^ = 31%) (Figure 4).

### Lowest SpO_2_ During the Procedure and Hypoxemia Duration

**Figure 5:**
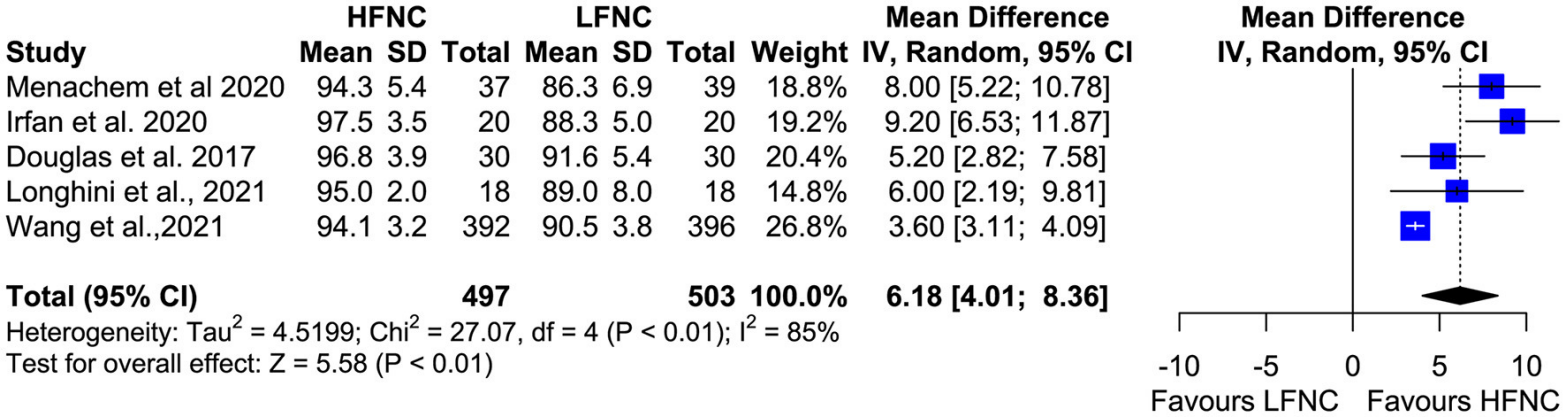
Meta-analysis of lowest Sp02 in patients receiving HFNC compared to LFNC in patients undergoing bronchoscopic procedures.

**Figure 6:**
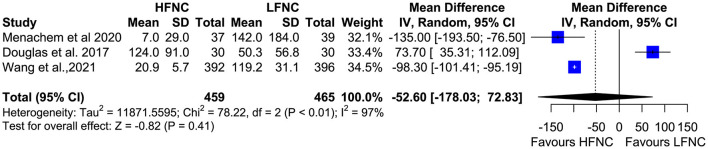
Meta-analysis of the duration of hypoxemia in patients receiving HFNC compared to LFNC in patients undergoing bronchoscopic procedures.

Five trials measured the lowest SpO_2_ value during bronchoscopy (11, 16, 17, 19, 20). Patients in the LFNC group had significantly lower SpO_2_ values than patients in the HFNC group in those receiving support with HFNC (MD = 6.18; 95% CI= 4.01, 8.36; *p* < 0.01; *I*^2^ = 85%) (Figure 5). Regarding hypoxemia duration, differences observed were not statistically significant. Two studies showed reduced hypoxemia duration when using HFNC (17, 20), and one study showed reduced hypoxemia duration when using LFNC (MD = −52.6; 95% CI = −178.03, 72.83; *p* = 0.41; *I*^2^ = 97%) (Figure 6).

### Bronchoscopic Duration and PCO_2_ at the End of the Procedure

**Figure 7:**
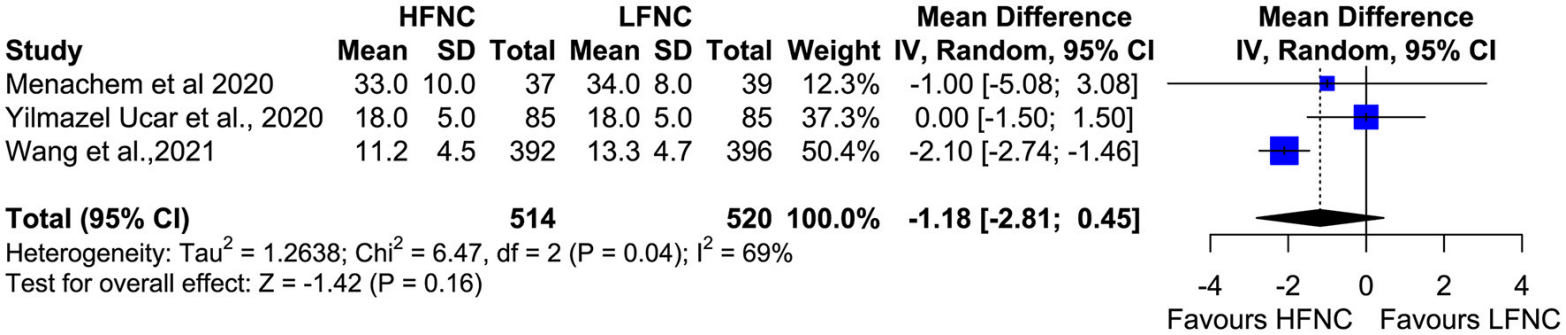
Meta-analysis of the duration of procedure in patients receiving HFNC compared to LFNC in patients undergoing bronchoscopic procedures.

**Figure 8:**
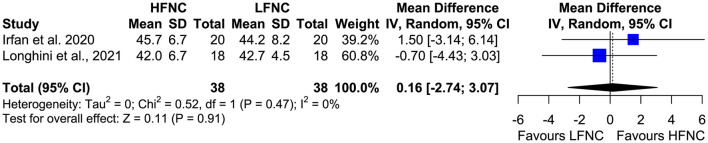
Meta-analysis of end procedural PCO2 in patients receiving HFNC compared to LFNC in patients undergoing bronchoscopic procedures.

Three trials measured procedure duration (17, 18, 20), and although the mean duration of bronchoscopy in the HFNC group was less than that in the LFNC group, the difference was not significant (MD = −1.18; 95% CI = −2.81, 0.45; *p* = 0.16; *I*^2^ = 69%) (Figure 7). Likewise, no difference was observed in two studies (16, 19) that measured PCO_2_ at the end of the procedure (MD = 0.16; 95% CI = −2.74, 3.07; *p* = 0.91; *I*^2^ = 0%) (Figure 8).

### SpO_2_, Respiratory Frequency, Heart Rate, and Mean Arterial Pressure 10 min After the Procedure

**Figure 9:**
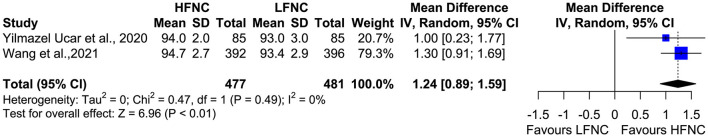
Meta-analysis of SpO2 10 min after the end of the procedure in patients receiving HFNC compared to LFNC in patients undergoing bronchoscopic procedures.

**Figure 10:**
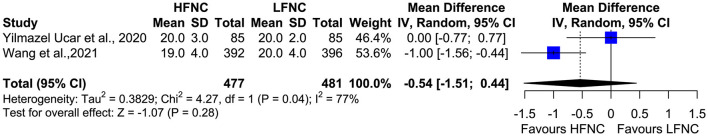
Meta-analysis of RF 10 min after the end of procedure in patients receiving HFNC compared to LFNC in patients undergoing bronchoscopic procedures.

**Figure 11:**
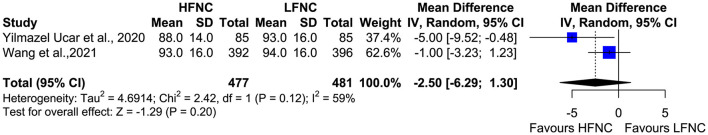
Meta-analysis of HR 10 minutes after the end of the procedure in patients receiving HFNC compared to LFNC in patients undergoing bronchoscopic procedures.

**Figure 12:**
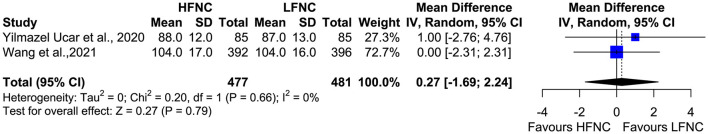
Meta-analysis of MAP 10 min after the end of procedure in patients receiving HFNC compared to LFNC in patients undergoing bronchoscopic procedures.

Two studies measured SpO_2_, heart rate (HR), respiratory frequency (RF), and mean arterial pressure (MAP) 10 min after the end of the procedure (18, 20). Mean SpO_2_ was significantly higher in the HFNC group (MD = 1.24; 95% CI = 0.89, 1.59; *p* < 0.01; *I*^2^ = 0%) (Figure 9). No difference was observed in RF (MD = −0.54; 95% CI = −1.51, 0.44; *p* = 0.28; *I*^2^= 77%; Figure 10), HR (MD = −2.5; 95% CI = −6.29, 1.3; *p* = 0.2; *I*^2^= 59%; Figure 11), and MAP (MD = 0.27; 95% CI = −1.69, 2.24; *p* = 0.79; *I*^2^ = 0%; Figure 12).

### Procedure Interruptions, Pneumothorax, and Intubation Incidence

**Figure 13:**
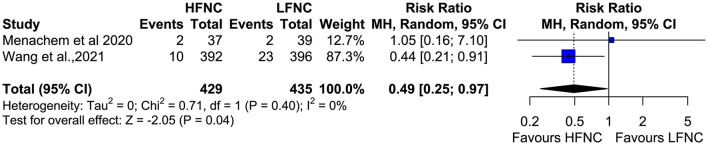
Meta-analysis of the rates of pneumothorax in patients receiving HFNC compared to LFNC in patients undergoing bronchoscopic procedures.

**Figure 14:**
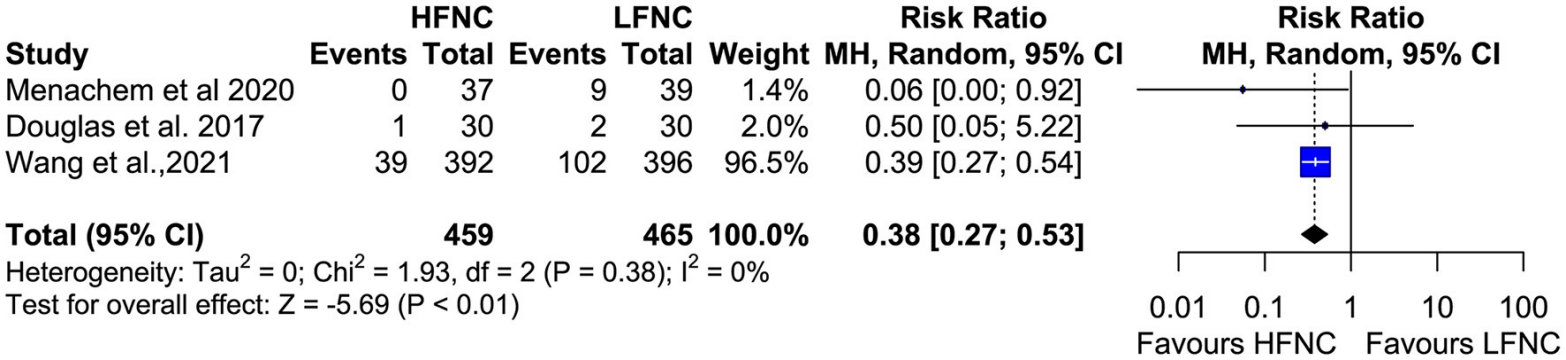
Meta-analysis of procedural interuptions in patients receiving HFNC compared to LFNC in patients undergoing bronchoscopic procedures.

Data on procedure interruptions were available in three trials (11, 17, 20). Bronchoscopy was less frequently interrupted in the HFNC group compared to the LFNC group (RR = 0.38; 95% CI = 0.27, 0.53; *p* < 0.01; *I*^2^ = 0%) (Figure 13). Pneumothorax incidence also differs significantly between the two groups and is more frequent in patients receiving support with LFNC (RR =0.49; 95% CI = 0.25, 0.97; *p* =0.04; *I*^2^= 0%) (Figure 14). Finally, in both arms, intubation incidence was zero in two trials that measured this outcome (16, 19).

## Discussion

The main outcome of our study was that the implementation of HFNC during bronchoscopic procedures, including EBUS, can potentially reduce hypoxemic events compared to that of LFNC. This is of major clinical interest, since bronchoscopic procedures are usually time-consuming and require deep levels of sedation, and thus, more prone to cause desaturations (21). Moreover, EBUS scopes have larger diameter and, with additional use of a balloon, can exert a greater negative effect on lung mechanics by increasing upper and lower airway resistance (18). HFNC can provide a CPAP effect on high-flow rates, humidification, and heating of inspired air, thus preventing alveolar collapse and lung de-recruitment while at the same time preserving lung compliance and upper airway patency, and washing out anatomic dead space by administering FiO2 up to 1. Therefore, it can protect from the development of hypoxemia during bronchoscopy procedures, most probably through preservation of normal lung mechanics (3, 5, 6). Of note, in at least three of the included studies, the CPAP effect of HFNC could be hampered, since the oral route was the preferred one for bronchoscopy. Therefore, the beneficial CPAP effect of HFNC could be greater if the nasal route was used (20). None of these beneficial effects of HFNC can be provided by any other conventional oxygenation method, where FiO2 is highly variable and affected by the breathing pattern of a patient while the CPAP effect is absent (3, 22).

In the LFNC group, hypoxemic events were recorded in a surprisingly high rate of 33.6% of procedures compared to 11.5% in the HFNC group (Figure 3). Desaturations and minimum SpO2 during bronchoscopy can increase the risk of post-procedural events like supraventricular arrythmias and persistent hypoxemias (7, 23). The type of procedure (BAL, forceps/transbronchial biopsy, EBUS), age of patient, use and type of sedation, and position of patient during bronchoscopy are related to peri- and post-procedural hypoxemia and cardiac arrythmias (7, 24). Cardiac complications, especially atrial and ventricular arrhythmias, are associated with minimum SpO2 value during the procedure and pose a great risk for post-procedural complications (25). Therefore, the utility and efficacy of oxygenation methods that can reduce peri- and post-procedural hypoxemia and related cardiac complications are of great importance. Indeed, our study showed that SpO_2_ 10 min post bronchoscopy was significantly lower in the HFNC group, which means that the patients under HFNC support recovered faster. Hypoxemia may persist for 2 h post bronchoscopy (26), and no study so far has examined the need for post bronchoscopy oxygen supplementation therapy (7). Therefore, the use of HFNC could reduce the need for post bronchoscopy oxygen supplementation therapy. No major differences were noted for HR, MAP, and RF.

Of note, in case of severe hypoxemia, the attending physician must access the airway, and in some cases reverse anesthesia and ventilate the patient to compensate hypoxemia and hypercarbia prolonging the duration of the procedure (23). Our study showed that procedural interruptions where indeed significantly less common in the HFNC group (11, 17, 20), and that the duration of both bronchoscopy and hypoxemia (SpO2 < 90%) and end-procedure PCO_2_ was longer in the LFNC group than in the HFNC group, but the differences were not significant (11, 16–20).

With regard to major post-procedural complications, none of the studies reported any bronchoscopy-related intubations or deaths. This observation can be explained by the fact that most of the patients enrolled in the studies were outpatients or inpatients with no major comorbidities, such as concurrent respiratory failure. Development of severe respiratory failure requiring intubation and mechanical ventilation during bronchoscopy is less common in outpatients (< 0.01%) (27, 28) than in inpatients. In the paper by Menachem et al. (17) authors reported higher satisfaction rates of the treating physicians in the HFNC group, evidence that reflects its beneficial effects. Pneumothorax incidence was reported in two studies (17, 20) with surprisingly significantly less events in the HFNC group. Even if HFNC administration has a limited CPAP effect, its application is related to increased respiratory support leading to less patient agitation, increased patient comfort, and, subsequently, better cooperation and shorter bronchoscopy duration (20, 29). No viral or bacterial contaminations were recorded.

Our study has many strengths and limitations. According to our knowledge, it is the biggest meta-analysis in the research field of bronchoscopy-induced hypoxemia, because it included a significant number of patients. All the included studies are RCTs with low heterogeneity and are of high quality. One primary and 10 secondary clinically relevantend points were addressed. Our study included almost 5 times more patients compared to an analogous meta-analysis by Su et al. (30). Therefore it had greater statistical power to address more clinically relevant outcomes like pneumothoraxes, mean SpO2, and other significant cardiorespiratory parameters in post bronchoscopy recovery time that have not been addressed in previous analogous studies (30). At the same time, our study showed significantly more procedure interruptions in the LFNC group, evidence that the study of Chien-Ling Su et al. failed to show, most probably because of the limited number of included patients (257) (30). Pelaia et al., in a recent systematic review and meta-analysis (31) addressed the benefits of HFNC vs. LFNC vs. continuous positive airway pressure (CPAP) modalities. They showed that HFNC is superior to LFNC with regard to lowest SpO2 and number of hypoxemic events, and that CPAP is superior to HFNC in the same clinical parameters. Nevertheless, our study focused solely on the benefit of HFNC vs. LFNC in bronchoscopic procedures, examining a multitude of clinically relevant outcomes in a much larger sample size.

Nevertheless, the exclusion criteria of all the studies were quite stringent, and patients with major comorbidities were not included, potentially affecting the generalizability of the meta-analysis outcomes. Indeed, all the studies were performed in a non-ICU setting, and the patients that were selected represent the bulk of the out-and inpatient population that would need to undergo bronchoscopy for various clinically significant diagnostic reasons like cancer, interstitial lung diseases, and infections. These underlying clinical conditions may compromise patients' periprocedural respiratory function. Unfortunately, in this meta-analysis, we cannot infer what the effects of HFNC would be in patients with severe respiratory failure and other major comorbidities who undergo bronchoscopy, but we can safely assume the inadequacy of low-flow systems during advanced bronchoscopic procedures in fragile populations with concurrent respiratory failure. Hypoxemia during bronchoscopy is more common in patients undergoing BAL, receiving sedation with benzodiazepines, and in those with compromised lung function (PEFR < 60% and FEV1 < 1 L) (32, 33). Of note, 51% of patients with FEV1 < 1,000 ml and 93% of those with FEV1 < 500 ml will develop hypoxemia during bronchoscopy (33). In recent real life studies (24, 34, 35), older age (> 70), inpatient status, duration of procedure, lower baseline SpO2, obstructive sleep apnea syndrome (OSAS), EBUS procedures and deep sedation were associated with higher complication rates and required escalation of care. Therefore, we can hypothesize that the benefits of HFNC application would be maximized in similar populations. No clear data exists examining the increased financial cost associated with the procurement of HFNC devises and consumables vs. possible benefits for the use of HFNC during bronchoscopy in the general population. We can assume that this can be compensated by reduction in the need for hospitalization and escalation of care, i.e., higher rates of pneumothoraxes in the LFNC group (Figure 13). Nevertheless, the use of HFNC in patients with severe respiratory failure and at risk for intubation has been proven to be cost-effective and safe (36).

Another limitation of our study is the variability of the respiratory support in both study arms (HFNC and LFNC) with s to devices/modalities, flow and FiO2, and variability in bronchoscopy route (oral vs. nasal) that might affect the effect of CPAP. The anesthetists and/or the bronchoscopists changed FiO2 during the procedure to match the patients' needs. As a result, the received FiO2 during desaturation event was variable, and a subgroup analysis matching the FiO2 was not feasible (37). In the majority of the included RCTs, the oral route was used; therefore, the beneficial effect of HFNC could be greater if the nasal route was preferred. The study population in the trial by Ben-Menachem et al. (17) differed from that of the other trials. Sedatives or anesthetic agents varied among the trials, and sedation was not used in all the studies (Table 1). With regard to hard outcomes like intubation rates and deaths, these were reported in only two RCTs. Of note, another limitation of our study is that the favorable effect of HFNC in reducing the rates of pneumothoraxes and procedural interruptions are driven mainly by the study of Wang et al. (20).

Better designed and larger multicenter randomized control trials are sorely needed, including patient-centered outcomes such as complications requiring hospitalization, intubation rates, prolonged post-procedural hypoxemic events, cost effectiveness calculations, and inclusion of more representative subpopulations such as those with severe comorbidities who need to undergo bronchoscopy.

## Conclusion

This meta-analysis highlights the potential superiority of HFNC to LFNC in reducing hypoxemic events and procedural interruptions during bronchoscopy. This potential protective effect of HFNC also extends to reduced rates of pneumothoraxes.

## Data Availability Statement

The raw data supporting the conclusions of this article will be made available by the authors, without undue reservation.

## Author Contributions

Data extraction was performed independently by two authors (FS and VK) while two authors (TK and AT) examined the two data sets for discrepancies. The rest of the authors provided expert insight for the physiology and the pathophysiology of NIV/high flow in bronchoscopies. GS provided insight for EBUS/bronchoscopies and their utility. All authors contributed to the article and approved the submitted version.

## Funding

Publication of this manuscript was financed by the Research Committee of the University of Patras.

## Conflict of Interest

The authors declare that the research was conducted in the absence of any commercial or financial relationships that could be construed as a potential conflict of interest.

## Publisher's Note

All claims expressed in this article are solely those of the authors and do not necessarily represent those of their affiliated organizations, or those of the publisher, the editors and the reviewers. Any product that may be evaluated in this article, or claim that may be made by its manufacturer, is not guaranteed or endorsed by the publisher.
